# CLINICIAN PERCEPTIONS OF SOCIAL WORKERS AND COMMUNITY HEALTH WORKERS IN PRACTICES CARING FOR FRAIL ELDERS

**DOI:** 10.1093/geroni/igz038.256

**Published:** 2019-11-08

**Authors:** Julie Berrett-Abebe, Karen Donelan, David I Auerbach, Peter Maramaldi, Barbara Berkman

**Affiliations:** 1 Massachusetts General Hospital, Boston, Massachusetts, United States; 2 Montana State University, Newton, Massachusetts, United States; 3 Simmons University, Boston, Massachusetts, United States; 4 Columbia University, Boston, Massachusetts, United States

## Abstract

Social workers (SW) and community health workers (CHW) have emerged as key workforce personnel in efforts to care for elders in the U.S. However, little is known about the presence and roles of these professionals in outpatient practices. This paper presents findings from a nationally representative survey of geriatrics and primary care practices. Key findings include: reported challenges in meeting the social service needs of elders, underutilization of SW, and fuller utilization of social work competencies in practices in which both SW and CHW were present. These findings offer a unique perspective of SW on interprofessional teams and have implications for the future of the profession.

